# Supplementary material for: CdS-Based Hydrothermal Photocatalysts for Complete Reductive Dehalogenation of a Chlorinated Propionic Acid in Water by Visible Light
Source: Nanomaterials (Basel). 2024 Mar 26;14(7):579. doi: 10.3390/nano14070579 (PMC11013931; doi:10.3390/nano14070579)
Supplement: Supplementary file 1 [file nanomaterials-14-00579-s001.zip › nanomaterials-2887544-supplementary.pdf]

nanomaterials

MDPI

Supplementary Matrials

CdS-Based Hydrothermal Photocatalysts for Complete Reductive Dehalogenation of a Chlorinated Propionic Acid in Water by Visible Light

Martina Milani [1,*], Michele Mazzanti [1], Claudia Stevanin [2], Tatiana Chenet [2], Giuliana Magnacca [3], Luisa Pasti [2,*] and Alessandra Molinari [1]

[1] Dipartimento di Scienze Chimiche, Farmaceutiche ed Agrarie, Università di Ferrara, Via Luigi Borsari 46, 44121 Ferrara, Italy
[2] Dipartimento di Scienze dell'Ambiente e della Prevenzione, Università di Ferrara, Corso Ercole I d'Este 32, 44121 Ferrara, Italy
[3] Dipartimento di Chimica, Università di Torino, Via P. Giuria 7, 10125 Torino, Italy
* Correspondence: martina.milani@unife.it (M.M.); luisa.pasti@unife.it (L.P.)

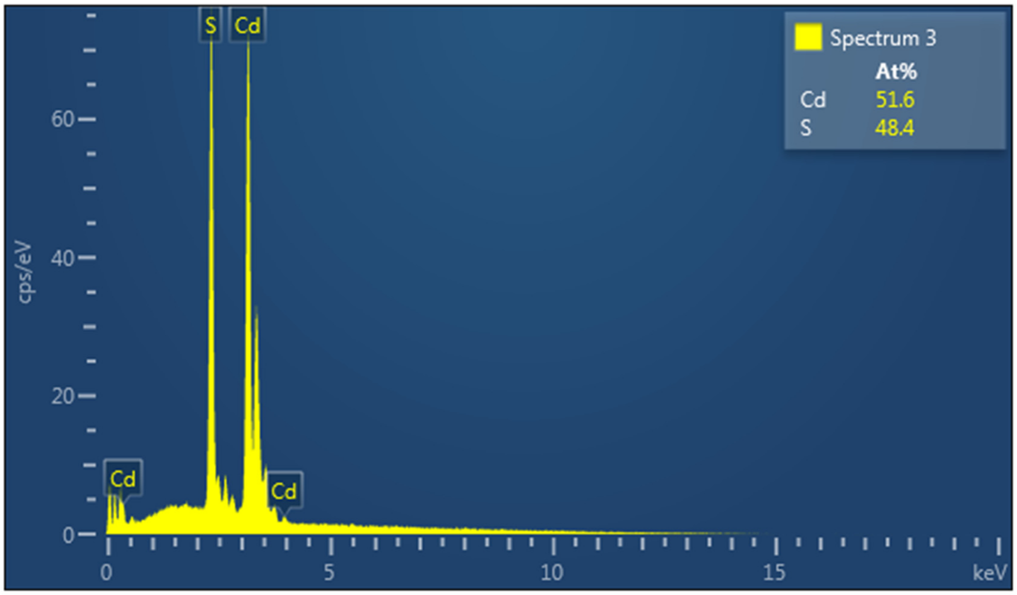

Figure S1. EDS pattern of CdS-HT.

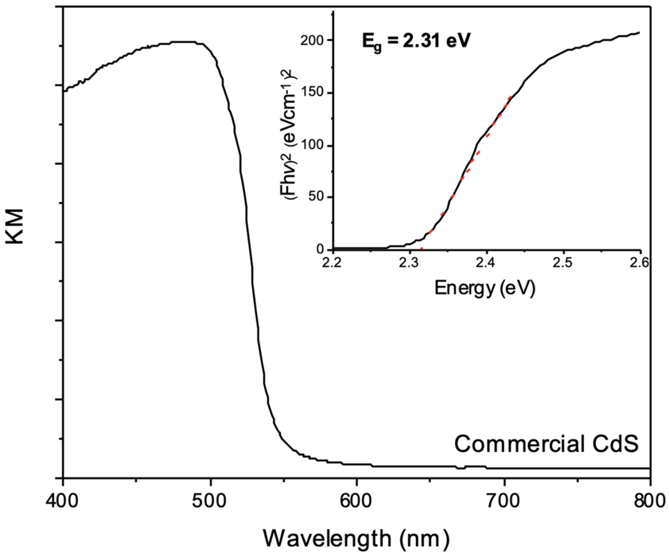

Figure S2. Absorption spectrum (KM units) of commercial CdS and Tauc plots in the inserts.

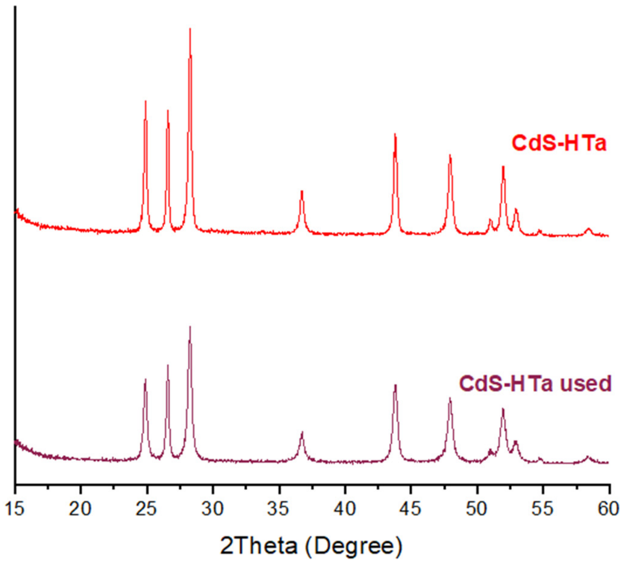

Figure S3. XRD patterns of CdS-HTa before and after irradiation.

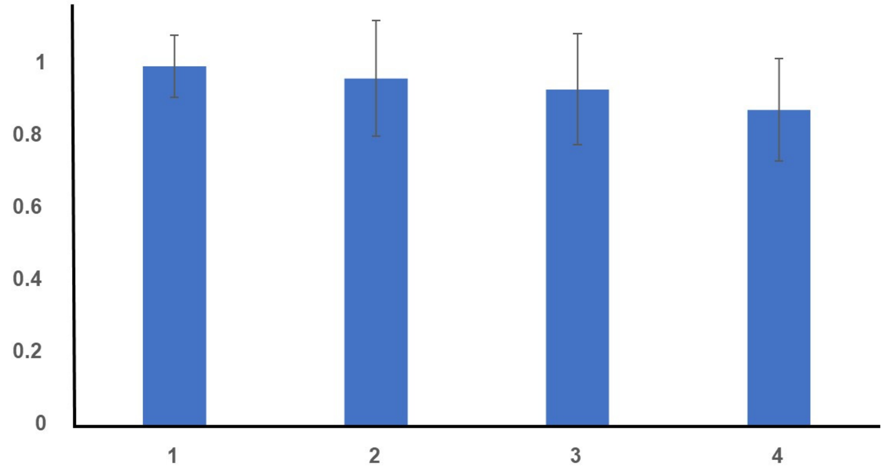

Figure S4. Photocatalyst recycling tests.

Experimental details of the use of powder oyster shells

Adsorbent preparation

One hundred oyster shells were collected from a shell disposal site in Sacca di Goro (Northern Adriatic Sea, Italy).

The shells were first brushed to remove any residual mollusk tissue, cleaned thoroughly with deionized MilliQ water (Millipore, MA, USA), and then dried in an oven at 50°C overnight.

A total of 30 shells were selected and milled using a grinder (Retsch, GmbH & Co., Germany) to obtain a fine powder.

Adsorption isotherm

An amount of 20 mL of Cd^{2+} solution was added to crimp-top reaction glass flasks sealed with PTFE septa (Supelco, Bellefonte, PA, USA) with 40 mg of shell powder. The initial concentrations of Cd^{2+} in the solutions were in the range of 1-15 mg/L. The equilibration time was 30 min, determined through uptake experiments. After 20 h of contact, longer than the equilibration time, under stirring (600 rpm) and at a controlled temperature of 38.4 ± 0.5°C, the adsorbent was separated from the solution by filtration using 25 mm syringe filters with PVDF membrane 0.45 µm (Agilent Technologies, Santa Clara, CA, USA), the pH of the solution was recorded for each solution after equilibration.

The concentration of Cd^{2+} in the solution, before (C_0) and after (C_e) the contact with the adsorbent material, was determined by ICP-MS, as described in the main text. The adsorbed quantity q (mg/g) was calculated by:

$$q = \frac{(C_0 - C_e) \cdot V}{m}$$

where V is the solution volume and m is the mass of the adsorbent. Fig. S5 shows the adsorption data.

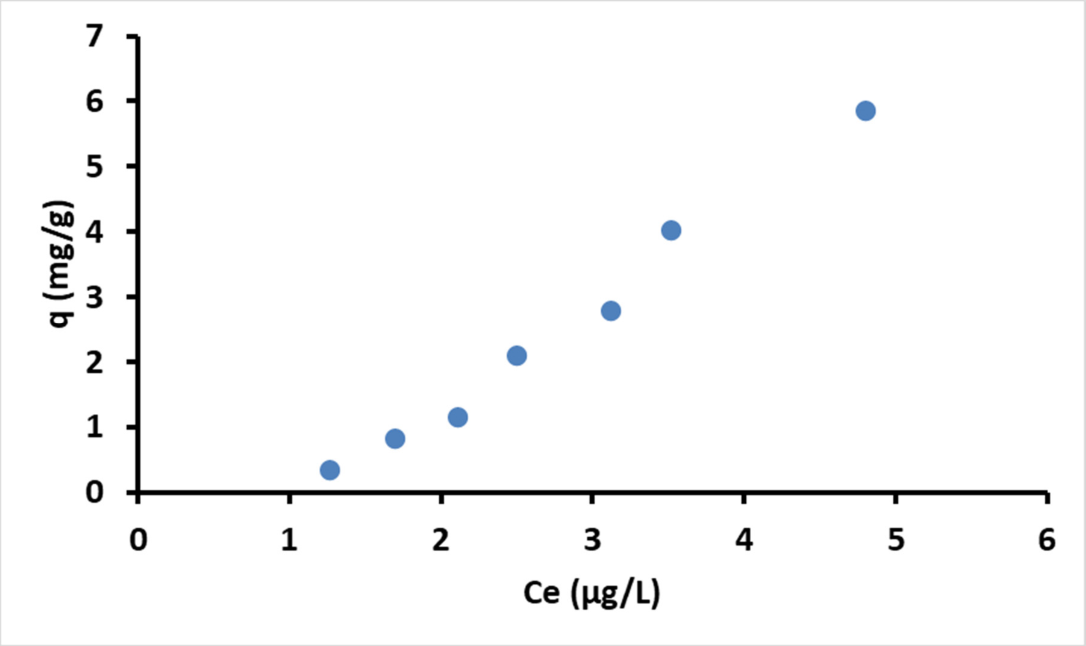

Figure S5. Adsorption isotherm of Cadmium onto oyster powdered shells.

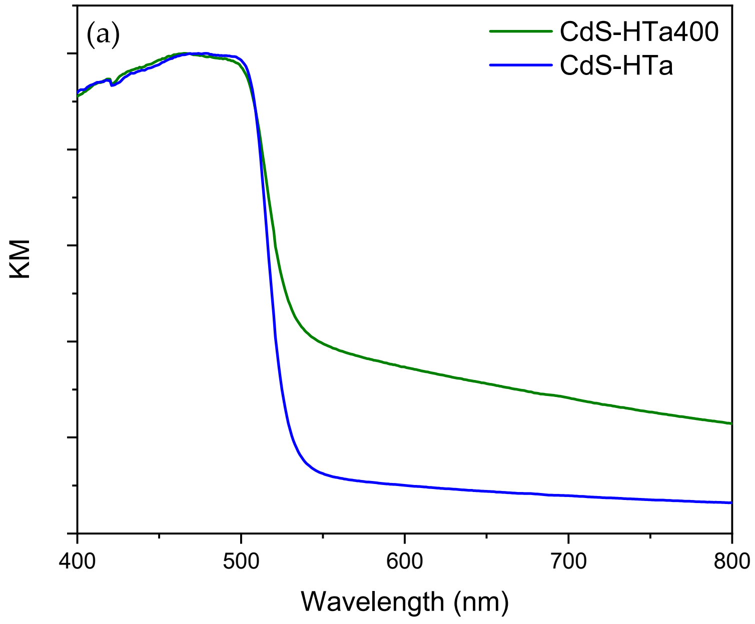

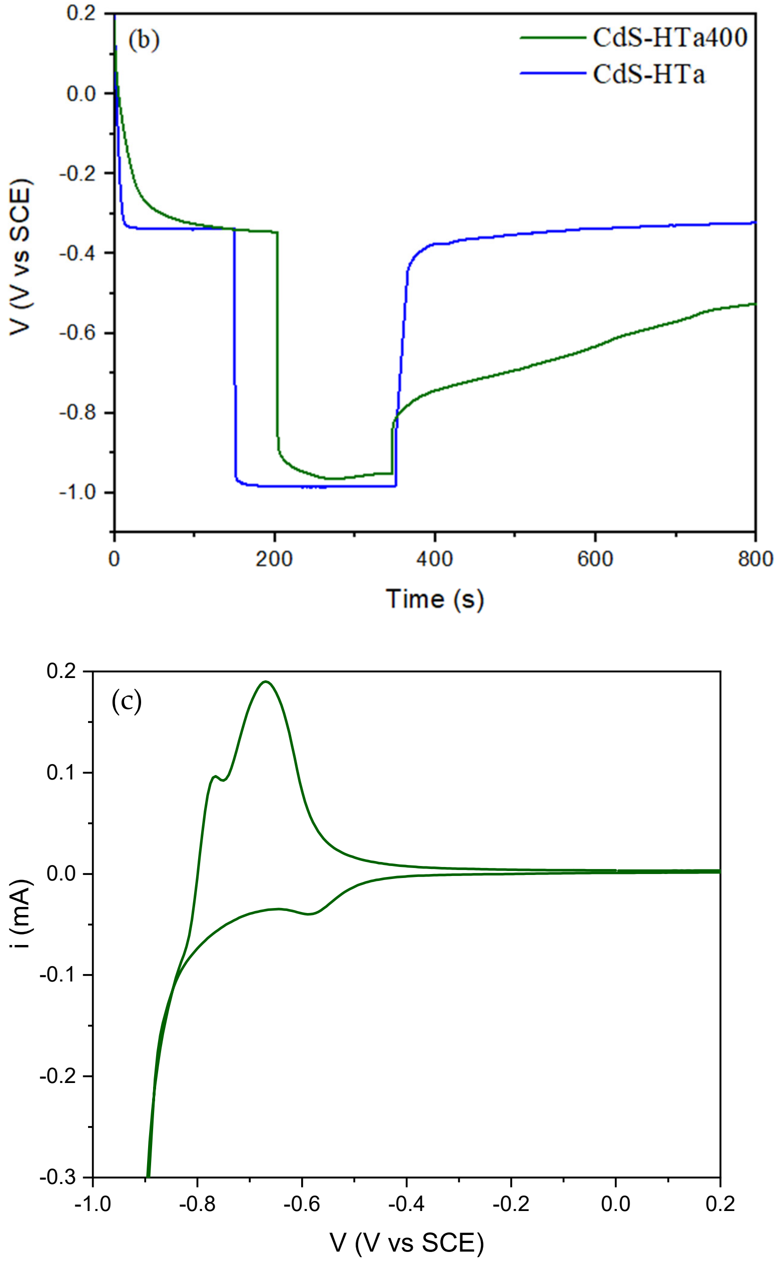

Figure S6. (a) Absorption spectra (KM units) of CdS-HTa (blue) and CdS-HTa400 (green) **(b)** Potential vs. time experiments of FTO/CdS-HTa (blue) and FTO-CdS-HTa400 (green) electrodes in deaerated aqueous solution containing HCOONa (1M) **(c)** Dark cyclic voltammetry (0.2V / 1.0V vs SCE) of FTO/CdS-HTa400 electrode in an aqueous solution containing HCOONa (1M).
